# Effect of primary care physicians' use of estimated glomerular filtration rate on the timing of their subspecialty referral decisions

**DOI:** 10.1186/1471-2369-12-1

**Published:** 2011-01-14

**Authors:** Raquel C Greer, Neil R Powe, Bernard G Jaar, Misty U Troll, L Ebony Boulware

**Affiliations:** 1 Division of General Internal Medicine, Johns Hopkins University School of Medicine, Baltimore, Maryland, USA; 2 Welch Center for Prevention, Epidemiology and Clinical Research, Baltimore, Maryland, USA; 3 Department of Medicine, University of California San Francisco, San Francisco, California, USA; 4 Department of Epidemiology, Johns Hopkins Bloomberg School of Public Health, Baltimore, Maryland, USA; 5 Division of Nephrology, Johns Hopkins University School of Medicine, Baltimore, Maryland, USA; 6 Nephrology Center of Maryland, Baltimore, Maryland USA

## Abstract

**Background:**

Primary care providers' suboptimal recognition of the severity of chronic kidney disease (CKD) may contribute to untimely referrals of patients with CKD to subspecialty care. It is unknown whether U.S. primary care physicians' use of estimated glomerular filtration rate (eGFR) rather than serum creatinine to estimate CKD severity could improve the timeliness of their subspecialty referral decisions.

**Methods:**

We conducted a cross-sectional study of 154 United States primary care physicians to assess the effect of use of eGFR (versus creatinine) on the timing of their subspecialty referrals. Primary care physicians completed a questionnaire featuring questions regarding a hypothetical White or African American patient with progressing CKD. We asked primary care physicians to identify the serum creatinine and eGFR levels at which they would recommend patients like the hypothetical patient be referred for subspecialty evaluation. We assessed significant improvement in the timing [from eGFR < 30 to ≥ 30 mL/min/1.73m^2^) of their recommended referrals based on their use of creatinine versus eGFR.

**Results:**

Primary care physicians recommended subspecialty referrals later (CKD more advanced) when using creatinine versus eGFR to assess kidney function [median eGFR 32 versus 55 mL/min/1.73m^2^, p < 0.001]. Forty percent of primary care physicians significantly improved the timing of their referrals when basing their recommendations on eGFR. Improved timing occurred more frequently among primary care physicians practicing in academic (versus non-academic) practices or presented with White (versus African American) hypothetical patients [adjusted percentage(95% CI): 70% (45-87) versus 37% (reference) and 57% (39-73) versus 25% (reference), respectively, both p ≤ 0.01).

**Conclusions:**

Primary care physicians recommended subspecialty referrals earlier when using eGFR (versus creatinine) to assess kidney function. Enhanced use of eGFR by primary care physicians' could lead to more timely subspecialty care and improved clinical outcomes for patients with CKD.

## Background

Chronic kidney disease (CKD) is a growing public health problem with over ten percent of United States (U.S.) adults having some form of kidney damage and/or decreased kidney function[1]. Patients with CKD are at increased risk of poor clinical outcomes including cardiovascular disease, hospitalizations, and death[2]. Timely referral of patients with CKD to specialist care has been shown to improve the morbidity and mortality associated with CKD[3,4]. Therefore, clinical practice guidelines recommend patients with CKD receive subspecialty referrals when their glomerular filtration rate is less than 30 mL/min/1.73m^2 ^to provide adequate time to manage the late complications of CKD and to prepare patients for renal replacement therapy[5-8]. Guidelines further advise referrals occur earlier (glomerular filtration rate greater than 30 mL/min/1.73m^2^) when patients show evidence of risk factors for rapid CKD progression, to exclude primary renal diseases when the etiology of CKD is unclear, and for the management of the early sequelae (including anemia and bone disease) of CKD, which are associated with poor clinical outcomes[5-8]. Despite these recommendations, primary care physicians, who care for a majority of the growing number of patients with stage 3 CKD, have been shown in multiple studies to have difficulties recognizing the severity of CKD, which may contribute to missed or late referrals[9-19].

Some primary care physicians' suboptimal recognition of the severity of CKD may be due, in part, to their use of serum creatinine to estimate kidney function, which is considerably less precise than more recently developed equation-based estimates of the glomerular filtration rate (eGFR)[20,21]. Studies suggest U.S. primary care physicians' use of eGFR has been widely variable[10,13,16-19]. Since its inception in 2002, the National Institutes of Health's National Kidney Disease Education Program (NKDEP) has encouraged clinical laboratories to automatically report eGFR along with patient laboratory results whenever a serum creatinine is ordered to facilitate the use of eGFR in clinical decision making for patients with CKD[22]. Although a majority of larger laboratories in the U.S. currently report eGFR (77%), less than half (38%) of all clinical laboratories nationwide calculate and report eGFR[22-24].

It is unknown whether the timing of U.S. primary care physicians' decisions to refer their patients with CKD to subspecialty care could be improved by their use of eGFR rather than serum creatinine to estimate CKD severity. Evidence of an effect of the use of eGFR on U.S. primary care physicians' CKD management decisions could influence the expansion of efforts to educate primary care providers regarding the use of eGFR to estimate CKD severity and to encourage clinical laboratories to automatically report eGFR to improve the care of patients with CKD. In a national study, we assessed the effect of U.S. primary care physicians' use of eGFR on the timing of their subspecialty referral decisions for patients with CKD.

## Methods

### Identification of Study Participants

As part of a national cross-sectional study conducted between August 2004 and August 2005 to assess U.S. physicians' clinical care of patients with CKD, we assessed whether primary care physicians' use of eGFR would affect the timing of their decisions to refer patients to CKD subspecialty care. We identified a random stratified sample of 400 family physicians, 400 internists, and 400 nephrologists using the American Medical Association Physician Master File and mailed physicians a self-administered questionnaire, which could be completed on paper or using the Internet. Physicians were ineligible for the study if they were not in active clinical practice or were not contactable through the 7 total mailings and 4 reminder telephone calls. Participating physicians were reimbursed $20. The Johns Hopkins Medicine Institutional Review Board approved the study protocol.

We asked physicians several questions regarding their preferred management of patients with progressing advanced CKD, including two questions to assess the threshold of kidney function at which they would refer patients for subspecialty care based on their use of either serum creatinine or eGFR to estimate kidney function. Of the 959 physicians in active clinical practice, a total of 126 nephrologists and 178 primary care physicians responded to the questionnaire. The intent of this analysis was to assess the effect of eGFR reporting on the timing of primary care physicians' recommendations for subspecialty referral. We therefore limited our study sample to the family physicians (n = 70) and internists (n = 84) who answered both referral threshold questions.

### Questionnaire Content

**Figure 1:**
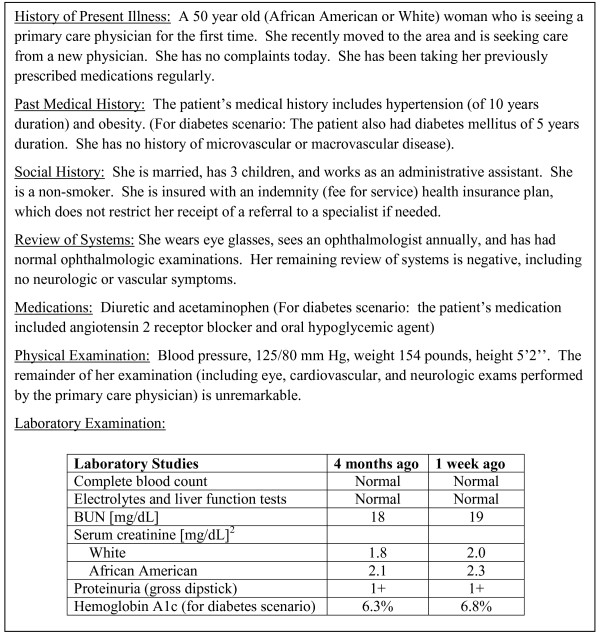
**Hypothetical case scenario**. We provided primary care physicians with one of four randomly assigned hypothetical scenarios, which varied on patient race (African American or White) and the presence or absence of diabetes. * Complete blood count includes hemoglobin, hematocrit, platelet count, and white blood cell count. ^† ^For the "4 month ago values", the estimated glomerular filtration rate (eGFR) for the White and African American patient was 32 mL/min/1.73m^2 ^and for the "1 week ago" values the eGFR was 28 and 29 mL/min/1.73m^2^, respectively (using the 4-variable Modification of Diet in Renal Disease Study equation)[20].

We provided primary care physicians with one of four randomly assigned hypothetical case scenarios featuring a 50 year-old female patient with hypertension and obesity being evaluated by a primary care physician for the first time. The hypothetical patient had advanced progressing CKD, which would warrant subspecialty referral based on U.S. and international subspecialty organization recommendations[5-8]. Case scenarios varied randomly on patient race (African American or White) and the presence or absence of diabetes. (Figure 1) The scenarios revealed only the serum creatinine levels of the hypothetical patient, as subsequent questions assessed physicians' abilities to estimate eGFR from information provided in the scenario[9]. Hypothetical patients' serum creatinines varied according to patient race to ensure all hypothetical patients' eGFRs (upon which clinical practice guidelines for referral are based) would be similar.

**Figure 2:**
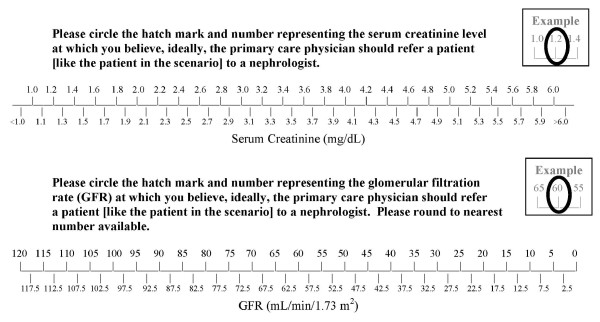
**Visual analog scales for serum creatinine and estimated glomerular filtration rate (eGFR) levels**. Physicians were asked to mark on the visual analog scales the serum creatinine level and the eGFR level at which they would recommend a primary care physician refer a patient, like the patient featured in the hypothetical scenario, for subspecialty care.

To assess the effect of primary care physicians' use of eGFR on the timing of their subspecialty referral decisions, we presented them with two visual analog scales, one featuring a range of numbers reflecting kidney function measured using serum creatinine [ranging from "<1.0 mg/dL" (better function) to ">6.0 mg/dL" (worse function)] and one featuring numbers reflecting kidney function measured using eGFR [ranging from 120 mL/min/1.73m^2 ^(better function) to 0 mL/min/1.73m^2 ^(worse function)]. We asked physicians to mark on the visual analog scales the serum creatinine level and the eGFR level at which they would recommend a primary care physician refer a patient, like the patient featured in the hypothetical scenario, for subspecialty care. (Figure 2)

### Assessment of Physician Characteristics

We assessed primary care physicians' demographic and practice characteristics [specialty (internists or family physician), practice type (academic or other), percent of time performing clinical duties, number of years in practice, ZIP code of practice, and participation in educational resources (attend conferences, read scientific journals, or other continuing medical education activities]. We also asked primary care physicians to select from a list the organizations they turn to for clinical practice guidelines as well as their awareness and compliance with guidelines regarding the referral of patients with CKD. We dichotomized the number of years in practice (≤ 10 years versus > 10 years) to reflect the approximate time interval in which physicians are required to enhance their clinical judgment and skills through the clinical practice board re-certification process[25,26]. Since most participating physicians spent the majority of their time performing clinical duties, we dichotomized their percent time performing clinical duties at the 25^th ^percentile (<80% versus ≥ 80%).

### Assessment of Timing of Primary Care Physicians' Subspecialty Care Referrals

We assessed differences in the timing of primary care physicians' subspecialty referrals based on their use of serum creatinine or eGFR by converting their selected serum creatinine values to eGFR using the Modification of Diet in Renal Disease Study equation[20]. We accounted for the randomly assigned race of hypothetical patients in the calculation of the creatinine-based eGFR. For each primary care physician, we calculated the absolute difference (in mL/min/1.73m^2^) between the eGFR and creatinine-based eGFR levels at which physicians recommended referral. The patient featured in the hypothetical scenario was at increased risk of rapid CKD progression and would therefore qualify for subspecialty referral before the eGFR reached 30 mL/min/1.73m^2 ^or less. We therefore considered primary care physicians' recommendations for subspecialty referral to have clinically significantly improved with their use of eGFR if their serum creatinine-based referral recommendations corresponded to an eGFR level of < 30 mL/min/1.73m^2^, but they recommended subspecialty referral at a level ≥ 30 mL/min/1.73m^2 ^when using eGFR.

### Statistical Analysis

We used bivariate (chi square, Wilcoxon rank-sum) analysis to assess differences in responding and non-responding physicians' number of years in practice and census region. We used Wilcoxon rank sum test to assess differences between the level of kidney function at which primary care physicians' recommended subspecialty referrals based on serum creatinine versus eGFR. We described the timing of primary care physicians' referrals (eGFR < 30 versus ≥ 30 mL/min/1.73m^2^) and the proportion of primary care physicians with clinically significant improvements in the timing of their referrals when using eGFR versus serum creatinine to estimate kidney function. We used multivariable logistic regression to identify physician (years in practice, practice setting, percent of time spent performing clinical duties, region of practice, and awareness of guidelines for referral) and hypothetical patient (diabetes presence and race) characteristics independently associated with clinically significant improvement in the timing of primary care physicians' referrals. We used Pearson's chi-square goodness of fit test to assess the model. We converted adjusted odds ratios to absolute probabilities and their corresponding 95% confidence intervals[27]. We performed all statistical analyses with STATA version 9.2 (Statacorp, College Station, Texas). The funders had no role in the conduct or conceptualization of this study.

## Results

### Study Participants

Of the 178 responding primary care physicians, 154 answered questions regarding the serum creatinine and eGFR level at which they would refer a patient, similar to the patient featured in the hypothetical scenario, for CKD subspecialty care. Responding and nonresponding primary care physicians did not differ in years in practice (median (interquartile range): 12 (3-21) versus 12 (5-20), respectively; p = 0.64) or census region of practice (Northeast, 25% versus 20%, Midwest, 24% versus 25%; South, 29% versus 33%; and West 22% versus 22%, respectively; p = 0.57).

A majority of primary care physicians practiced in non-academic settings, practiced greater than 10 years, spent greater than 80% of their time performing clinical duties, and reported attending conferences or reading scientific journals as an educational resource. Primary care physicians most frequently reported turning to internal medicine or non-nephrology specialty organizations for clinical practice guidelines, while fewer reported turning to United States Preventive Services Task Force or to nephrology organizations. One third of primary care physicians reported they were aware of subspecialty referral guidelines for patients with CKD, and a majority of these physicians (84%) reported following these guidelines in their own practices. Over half of primary care physicians were presented with a hypothetical case scenario featuring an African American patient or a patient with diabetes. Primary care physicians were equally distributed among census regions. (Table 1)

**Table 1:** Primary care physician and scenario characteristics

Physician characteristics	All N (%) N = 154
Physician Specialty	

Internal medicine	84 (55)

Family physician	70 (45)

Years in practice:	

≤ 10 years	76 (49)

>10 years	78 (51)

Practice type:	

Academic	25 (16)

Other	128 (84)

Percent clinical time:	

<80%,	23 (15)

≥ 80%	131 (85)

Census region:	

Northeast	40 (26)

Midwest	33 (21)

South	45 (29)

West	36 (23)

Aware of referral guidelines	

Yes	49 (32)

No	103 (68)

Educational resources:	

Conferences	134 (88)

Scientific journals	144 (95)

Other	78 (53)

Guideline organizations:	

Nephrology	46 (29)

Internal medicine	118 (77)

USPSTF	77 (50)

Specialty	101 (66)

Clinical scenario:	

Patient race: African American	84 (55)

Diabetes	89 (58)

Note. Due to missing values, categorical frequencies may not equal column total. For educational resources and guideline organizations, selections were not mutually exclusive.

ZIP code used for census region.

### Effect of eGFR on the Timing of Primary Care Physicians' Subspecialty Referrals

**Figure 3:**
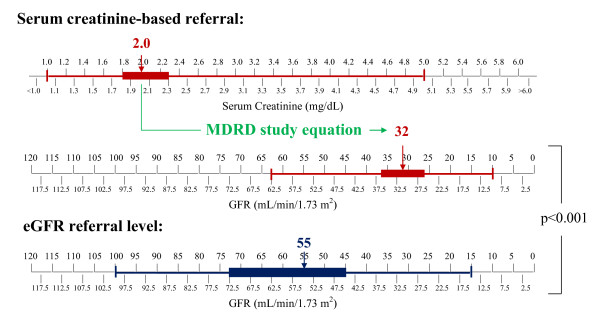
**Recommended serum creatinine and estimated glomerular filtration rate (eGFR)-based referral levels by primary care providers**. Note. Lines and bars represent the range and interquartile range of selected values, respectively. Abbreviations. eGFR, estimated glomerular filtration rate

Primary care providers recommended patients for subspecialty care earlier when using eGFR compared to when they used serum creatinine to estimate kidney function. The median serum creatinine level (interquartile range (IQR)) at which primary care physicians recommended subspecialty referral was 2.0 (1.8-2.3) mg/dL, corresponding to a median eGFR of 32 (26-36) mL/min/1.73m^2^) while the median eGFR level at which primary care physicians recommended subspecialty referral was 55 (45-72) mL/min/1.73m^2 ^(p < 0.001). (Figure 3) The median (IQR) absolute difference between eGFR and creatinine-based eGFR referral levels was 23 (12-40) mL/min/1.73m^2^. The improvement in timing of referrals with the use of eGFR was greater for physicians presented with a White hypothetical patient compared to physicians presented with an African American patient. There were no differences in the timing of referrals based on physicians' use of eGFR according to patients' presence or absence of diabetes. (Table 2)

**Table 2:** Recommended serum creatinine and estimated glomerular filtration rate (eGFR)-based referral levels by characteristics of the hypothetical patient

	Median referral level mL/min/1.73m^2^					
	Creatinine-based eGFR*	p value	eGFR	p value	Absolute difference	p value

Race		<0.001		0.47		0.03

African American	34		54		19	

White	28		55		26	

Diabetes		0.90		0.42		0.34

Yes	32		60		26	

No	32		50		22	

*The recommended median serum creatinine at referral for all versions of the clinical scenario was 2 mg/dL

When using eGFR to estimate kidney function, nearly all (94%) primary care physicians' recommended referrals when eGFR was ≥ 30mL/min/1.73m^2 ^compared to 55% of primary care physicians' making similar recommendations when using serum creatinine. Over a third of primary care physicians (40%) significantly improved the timing of their referrals when basing their recommendations on eGFR. In multivariable models, improved timing of referral recommendations was more prevalent among primary care physicians practicing in academic (versus non-academic) practices and physicians presented with hypothetical scenarios featuring a White (versus an African American) patient. (Table 3)

**Table 3:** Percent of primary care physicians with clinically significant improvement in the timing of their subspecialty referral recommendations with the use of estimated glomerular filtration rate (eGFR) by physician and scenario characteristics

Physician characteristics	% of primary care physicians with clinically significant improvement in their subspecialty referral recommendations^*^			
	
	Unadjusted	p value	**Adjusted**^† ^**(95% CI)**	p value
Years in practice:				

>10 years	44	0.31	50 (32-68)	0.11

≤ 10 years	36		36 (Ref)	

Practice type:				

Academic	56	0.07	70 (45-87)	0.01

Other	37		37 (Ref)	

Percent clinical time:				

<80%,	26	0.15	23 (8-49)	0.13

≥ 80%	42		42 (Ref)	

Census region:				

Midwest	33	0.65	38 (18-64)	0.47

South	38		40 (20-64)	0.56

West	39		41 (20-66)	0.60

Northeast	48		48 (Ref)	

Aware of referral guidelines:				

Yes	29	0.06	29 (15-48)	0.09

No	45		45 (Ref)	

Clinical scenario:				

Patient race:				

White	57	<0.001	57 (39-73)	<0.001

African American	25		25 (Ref)	

Diabetes				

Yes	43	0.36	42 (26-61)	0.44

No	35		35 (Ref)	

Note. A total of 152 participants with complete data were included in the model

*Clinically significant improvement in subspecialty referral recommendations was defined as present, if primary care physicians selected a serum creatinine-based referral level corresponding to an estimated glomerular filtration rate (eGFR) level of < 30 mL/min/1.73m^2^, but recommended subspecialty referral at level of ≥ 30 mL/min/1.73m^2 ^with the use of eGFR.

^†^Adjusted for all variables in the table.

## Discussion

In this national study, U.S. primary care physicians referred patients for subspecialty care earlier when they based their referral decisions on patients' eGFR compared to when they based their referral decisions on serum creatinine. Over a third of primary care physicians significantly improved the timing of their decisions with the use of eGFR. Improved timing of referrals was greater among primary care physicians practicing in academic settings and presented with White hypothetical patients. These findings provide insight regarding the potential impact of clinical laboratories' automatic reporting of eGFR on clinical care and patient outcomes.

To our knowledge, this is the first U.S. study to demonstrate the effect of primary care physicians' use of eGFR on the timing of their subspecialty referral decisions. Prior non-US observational studies investigating the association of eGFR reporting with CKD specialty referral practices were limited by their inability to account for various clinical and non-clinical policy and resource trends which could have also impacted referral practices[28-34]. Our study was designed to directly assess the impact of the use of eGFR on physician decision making under the same patient, provider, and system level influences. Our findings provide evidence that encouraging use of eGFR by primary care providers to assess kidney function and more widespread automatic reporting of eGFR by clinical laboratories could significantly improve the quality of care and clinical outcomes for patients with CKD by directly affecting physicians' clinical decisions. Earlier referrals to subspecialty care for the roughly one million U.S. adults with advancing CKD (defined as National Kidney Foundation Kidney Disease Outcome Quality Initiative stages 3 and 4 CKD with gross proteinuria) could impact several aspects of clinical care for these patients, including allowing for appropriate dosing of medications to accommodate impaired renal function, earlier avoidance of nephrotoxins which could hasten CKD progression, achievement of CKD directed blood pressure and lipid targets, treatment of early metabolic complications of CKD, as well as earlier preparation for renal replacement therapy, all of which have been recommended and many of which have been demonstrated to improve clinical outcomes[1,3,7,35-43].

There was substantial variation in the levels of eGFR at which U.S. primary care physicians recommended referral (ranging from eGFR of 15 to 100 mL/min/1.73m^2^), suggesting refinement of clinical practice guidelines to clarify the indications for referral may be needed. While very early referrals may be appropriate for patients with gross proteinuria or rapidly declining kidney function, very early referrals among some patients with less risk of progression (e.g. elderly persons with reduced but relatively stable kidney function) may be inappropriate with regard to resource utilization and availability of nephrologists[44,45]. Guidelines' clarification of clinical circumstances requiring more urgent referrals, as well as dissemination of these recommendations, may provide primary care physicians with greater confidence to care for the growing number of patients with CKD.

Differences in serum creatinine based on patient race and gender are well-established (with greater serum creatinine levels among men and African Americans)[46]. The extent to which our finding of physicians' greater improvement in the timing of referrals among Whites compared to African Americans reflects race-based inequities in care is unclear. Since we presented each physician with only one hypothetical patient scenario (featuring a patient of either White or African American race), we were unable to ascertain whether individual physicians' practice patterns would have changed if they saw patients of different races. It is highly possible later referrals of Whites based on serum creatinine reflects primary care physicians' lack of knowledge regarding the severity of kidney dysfunction associated with lower serum creatinine levels among Whites. In light of previous research demonstrating Blacks are more likely to receive later subspecialty referrals compared to Whites[4], our findings of equal referral timing among African Americans and Whites when primary care physicians used eGFR to estimate kidney dysfunction provides some reassurance that the use of eGFR may help narrow race-based differences in the timing of subspecialty referrals. The extent to which physicians' enhanced use of eGFR could narrow racial disparities in the long-term clinical outcomes of patients with CKD merits further study.

There are limitations of this study. First, physicians' recommendations for referral based on a hypothetical case scenario may not reflect their real practice patterns. Further, serum creatinines provided in the scenario might have caused an anchoring effect for the respondents when providing recommendations for referral based on serum creatinine. However, the use of a hypothetical case allowed us to assess physicians' decision-making regarding referral under similarly realistic conditions, and the inclusion of serum creatinine in the scenario would not have impacted within-individual-physician differences in their referrals when using serum creatinine versus eGFR. Second, we did not assess the rationale for the timing of physicians' referrals, which may have been based on factors other than the eGFR (i.e. presence of proteinuria or diabetes). Third, the study sample size was small, and primary care physicians' response rate was limited, possibly limiting our ability to detect all significant associations and the generalizability of our findings. Nonetheless, participating physicians practiced in several regions of the U.S. and in a variety of practice settings enhancing our ability to identify physician characteristics associated with improvement in the timing of referrals. Finally, the cross-sectional design of our study limits our ability to assess the potential long-term effects of physicians' eGFR referral decisions on patients' clinical outcomes. However, extensive research documenting poor clinical outcomes for patients experiencing late referrals for subspecialty care supports the potential significance of our findings[3,4].

## Conclusions

In conclusion, we found U.S. primary care physicians recommended subspecialty referrals earlier when assessing the severity of kidney dysfunction using eGFR compared to their assessments using serum creatinine. Physicians practicing in academic primary care settings and reviewing a hypothetical scenario featuring a White patient were more likely to improve the timing of their referral recommendations when using eGFR. Increased use of eGFR by primary care providers, in addition to more wide spread automated reporting of eGFR by U.S. clinical laboratories could improve the timing of subspecialty care and clinical outcomes for patients with CKD. Race-based differences in improved timing of referral with the use of eGFR warrant further study.

## Competing interests

The authors declare that they have no competing interests.

## Authors' contributions

RG conceived of the study, performed the statistical analysis and drafted the manuscript. EB participated in the design and coordination of the study and assisted with drafting the manuscript. RG, NP, and MT participated in the design of the study and MT participated in coordination of the study. All authors revised the manuscript, provided intellectual content to the work, and have read and approved the final manuscript.

## Pre-publication history

The pre-publication history for this paper can be accessed here:

http://www.biomedcentral.com/1471-2369/12/1/prepub
